# Xenon Reduces Neuronal Hippocampal Damage and Alters the Pattern of Microglial Activation after Experimental Subarachnoid Hemorrhage: A Randomized Controlled Animal Trial

**DOI:** 10.3389/fneur.2017.00511

**Published:** 2017-09-27

**Authors:** Michael Veldeman, Mark Coburn, Rolf Rossaint, Hans Clusmann, Kay Nolte, Benedikt Kremer, Anke Höllig

**Affiliations:** ^1^Department of Neurosurgery, RWTH Aachen University Hospital, Aachen, Germany; ^2^Department of Anesthesiology, RWTH Aachen University Hospital, Aachen, Germany; ^3^Department of Neuropathology, RWTH Aachen University Hospital, Aachen, Germany

**Keywords:** subarachnoid hemorrhage, early brain injury, animal model, xenon, neuroprotection

## Abstract

**Objective:**

The neuroprotective properties of the noble gas xenon have already been demonstrated using a variety of injury models. Here, we examine for the first time xenon’s possible effect in attenuating early brain injury (EBI) and its influence on posthemorrhagic microglial neuroinflammation in an *in vivo* rat model of subarachnoid hemorrhage (SAH).

**Methods:**

Sprague-Dawley rats (*n* = 22) were randomly assigned to receive either Sham surgery (*n* = 9; divided into two groups) or SAH induction *via* endovascular perforation (*n* = 13, divided into two groups). Of those randomized for SAH, 7 animals were postoperatively ventilated with 50 vol% oxygen/50 vol% xenon for 1 h and 6 received 50 vol% oxygen/50 vol% nitrogen (control). The animals were sacrificed 24 h after SAH. Of each animal, a cerebral coronal section (−3.60 mm from bregma) was selected for assessment of histological damage 24 h after SAH. A 5-point neurohistopathological severity score was applied to assess neuronal cell damage in H&E and NeuN stained sections in a total of four predefined anatomical regions of interest. Microglial activation was evaluated by a software-assisted cell count of Iba-1 stained slices in three cortical regions of interest.

**Results:**

A diffuse cellular damage was apparent in all regions of the ipsilateral hippocampus 24 h after SAH. Xenon-treated animals presented with a milder damage after SAH. This effect was found to be particularly pronounced in the medial regions of the hippocampus, CA3 (*p* = 0.040), and dentate gyrus (DG *p* = 0.040). However, for the CA1 and CA2 regions, there were no statistical differences in neuronal damage according to our histological scoring. A cell count of activated microglia was lower in the cortex of xenon-treated animals. This difference was especially apparent in the left piriform cortex (*p* = 0.017).

**Conclusion:**

In animals treated with 50 vol% xenon (for 1 h) after SAH, a less pronounced neuronal damage was observed for the ipsilateral hippocampal regions CA3 and DG, when compared to the control group. In xenon-treated animals, a lower microglial cell count was observed suggesting an immunomodulatory effect generated by xenon. As for now, these results cannot be generalized as only some hippocampal regions are affected. Future studies should assess the time and localization dependency of xenon’s beneficial properties after SAH.

## Introduction

### Background

Aneurysmal subarachnoid hemorrhage (SAH) is a subtype of stroke occurring at a relative young age causing either death or disability in many patients (1). Few people recover without impairments (2). The annual incidence is estimated to be about 9.1 patients per 100,000 (3). In around 85% of spontaneous SAHs, the underlying cause is the rupture of a cerebral aneurysm (4). The resulting increase in intracranial pressure (ICP), disruption of the blood–brain barrier and global ischemia contribute to early brain injury (EBI) (5). These injuries within the first 72 h of the initial ictus account for the later development of vasospasm and delayed cerebral ischemia (6). There is a substantial interest in EBI and in ways to reduce initial cerebral damage and thus indirectly attenuate secondary injuries.

The neuroprotective effect of the noble gas xenon has been well established in animal experiments for focal and global cerebral ischemia (7–11). Xenon has a proven additive neuroprotective effect to hypothermia in models of neonatal asphyxia (12–14). So far, xenon treatment has not been examined in the context of SAH. Multiple animal models of SAH have been established. The two most commonly used are the cisternal injection model and the endovascular perforation model (15). We opted for the latter as it better mimics the pathophysiology of an aneurysm rupture, and it is probably more suitable to investigate EBI (16). In this trial, the neuroprotective properties of the noble gas xenon were examined in the early phase after SAH using an endovascular perforation rat model. Despite its cost, xenon has demonstrated minimal side effects in extensive anesthesia studies, making it an interesting future treatment in human trials aiming for neuroprotection (17–21). Furthermore, xenon has already been approved in Europe for use as a general anesthetic.

## Materials and Methods

### Study Design

We performed a randomized four group controlled animal trial examining the neuroprotective effects of xenon inhalation (50 vol% for 1 h) with treatment initiation 1 h after SAH induction.

### Ethical Statement

The study protocol was approved by the government agency for animal use and protection (Protocol number: TVA 10416G1 approved by “Landesamt für Natur, Umwelt und Verbraucherschutz NRW,” Recklinghausen, Germany), all experiments were conducted in accordance with the Guide for Care and Use of Laboratory Animals (National Research Council, and the Committee for the Update of the Guide for the Care and Use of laboratory Animals; 8th edition 2011).

### Animals

**Figure 1:**
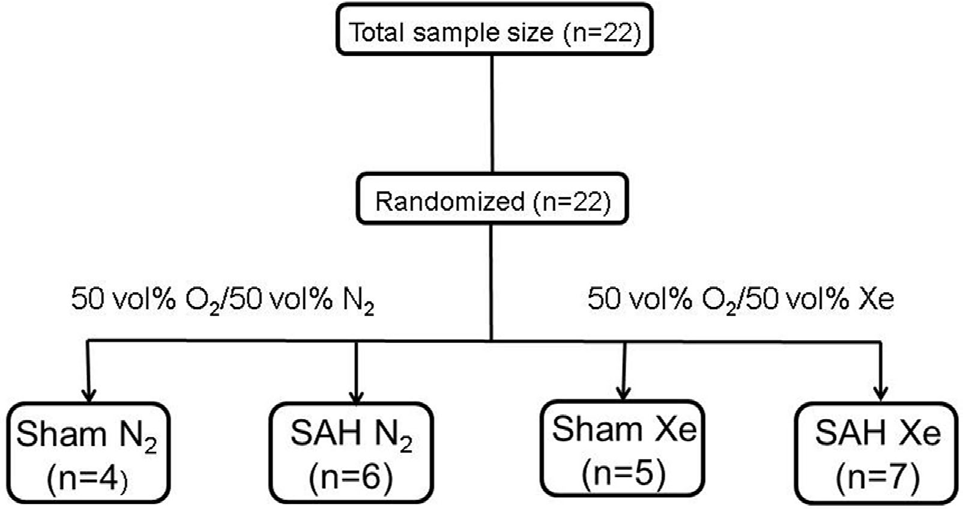
Flow chart of included animals. Animals were randomized by lot drawing in one of four groups. Sham N_2_ (Sham surgery after 1 h delay followed by ventilation with 50 vol% O_2_/50 vol% N_2_ for 1 h), Sham Xe (Sham surgery after 1 h delay followed by ventilation with 50 vol% O_2_/50 vol% Xe for 1 h), SAH N_2_ (SAH induction after 1 h delay followed by ventilation with 50 vol% O_2_/50 vol% N_2_ for 1 h), and SAH Xe (SAH induction after 1 h delay followed by ventilation with 50 vol% O_2_/50 vol% Xe for 1 h).

Male Sprague-Dawley rats (body weight 300–400 g, Charles River, Sulzfeld, Germany) were housed for at least 1 week before surgery with free access to food in a specific pathogen-free environment maintaining a 12-h light/dark cycle. Prior to anesthesia induction, animals were randomly assigned to one of the following four groups by lot drawing: Sham N_2_ (Sham surgery after 1 h delay followed by ventilation with 50 vol% O_2_/50 vol% N_2_ for 1 h), Sham Xe (Sham surgery after 1 h delay followed by ventilation with 50 vol% O_2_/50 vol% Xe for 1 h), SAH N_2_ (SAH induction after 1 h delay followed by ventilation with 50 vol% O_2_/50 vol% N_2_ for 1 h), and SAH Xe (SAH induction after 1 h delay followed by ventilation with 50 vol% O_2_/50 vol% Xe for 1 h) (see Figure 1).

### Experimental Procedure

Anesthesia was induced by intraperitoneal injection of a mixture of midazolam (2 mg/kg), medetomidine (0.15 mg/kg), and fentanyl (0.0075 mg/kg) (22, 23). A quarter of the initial dosage was injected in 30–45 min intervals to maintain anesthesia. Postoperative analgesia was started directly after surgery *via* intramuscular injection of metamizole (20 mg/kg) and continued until euthanasia (24 h after SAH induction). Animals were intubated using an 18 gauge i.v. cannula. Blood pressure was monitored by cannulation of the tail artery, electrolytes, and blood gases were monitored by repeated arterial blood gas analysis and body temperature was maintained at 37°C via a heating pad (Physitemp Instruments, Inc., Clifton, NJ, USA). After anesthesia induction, two laser Doppler flowmetry probes were fixated in proximity of the bregma to measure regional cerebral blood flow (rCBF), as previously described (Moor Instruments, Axminster, Devon, UK) (23). A left side parietal ICP probe was inserted for continuous ICP monitoring (Microsensor/Codman ICP Express Monitor, Codman/De Puy, Raynham, MA, USA). Baseline recordings of blood pressure, bilateral rCBF, heart rate, and ICP were done prior to surgery, during intervention, and 90 min thereafter (PowerLab, ADInstruments, Spechbach, Germany). SAH was induced by the polypropylene monofilament perforation technique initially described by Bederson et al. and modified by Veelken et al. (24, 25). The procedure was performed as previously described (26). After exposing the left common carotid artery, the left internal carotid artery (ICA) was identified and a 3–0 polypropylene suture with a diameter ranging from 200 to 250 µm (Prolene suture, Ethicon Inc., Somerville, NJ, USA) was advanced intravascularly. Perforation of the vessel and subsequent SAH was verified by a sudden increase in ICP and a bilateral decrease in rCBF. Sham-operated animals underwent the same anesthesia and surgical procedure, but the monofilament was advanced into the ICA without perforation of the vessel.

One hour after SAH induction or Sham surgery, the animals were ventilated for 1 h with either a mixture of 50 vol% O_2_/50 vol% xenon (Air Liquide, Paris, France) or 50 vol% O_2_/50 vol% N_2_ (control group). After treatment, anesthesia was stopped and animals were allowed to recover spontaneously. Analgesic treatment with metamizole (20 mg/kg intramuscular application every 8 h) was carried on until euthanasia (24 h after SAH induction). Euthanasia was performed 24 h after SAH induction by exsanguination under deep anesthesia followed by decapitation. Brains were harvested and cut into 2 mm coronal slices, fixated in paraformaldehyde, and embedded in paraffin.

### Histology/Immunohistochemistry

Sections of 2 µm thickness were cut from the paraffin-embedded brain slices and placed on silane-coated slides. Of every animal, the same section 3.60 mm posterior to the bregma was searched based on anatomical landmarks. After deparaffinization, a section was routinely hematoxylin/eosin (H&E) stained. Two consecutive sections were de-waxed, rehydrated, and heated in citrate buffer for antigen retrieval. After blocking of non-specific binding by incubation in PBS containing 2% normal goat serum, one slide per animal was incubated for 1 h with anti-NeuN (Millipore, MA, USA) as primary antibody diluted in blocking solution and one slide with anti-Iba-1. Appropriate biotinylated secondary antibodies were used (1:200, Vector Laboratories Ltd., Peterborough, UK) for 15 min, followed by DAB visualization (DAKO, Carpinteria, CA, USA). Appropriate negative controls without the primary antibodies were performed.

### Neuronal Cell Damage

**Figure 2:**
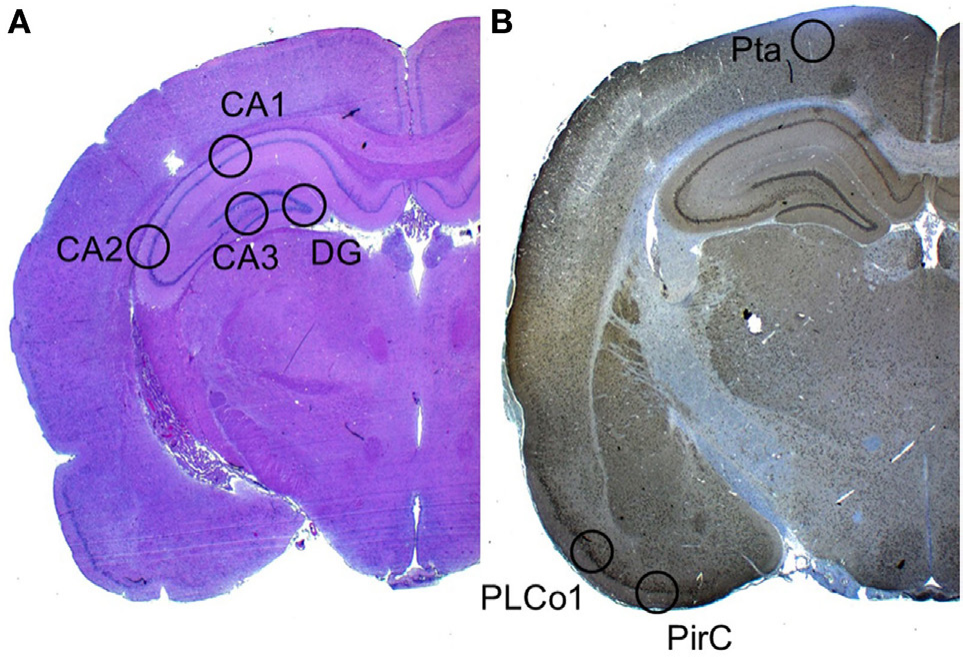
Selected regions of interest. **(A)** In H&E staining, a high power field was focused on four regions of interest: CA1, CA2, CA3, and DG. **(B)** In Iba-1 staining (the image displays a NeuN staining), a high power field was focused on the three cortical regions of interest: Pta, PLCo1, and Pir1.

**Figure 3:**
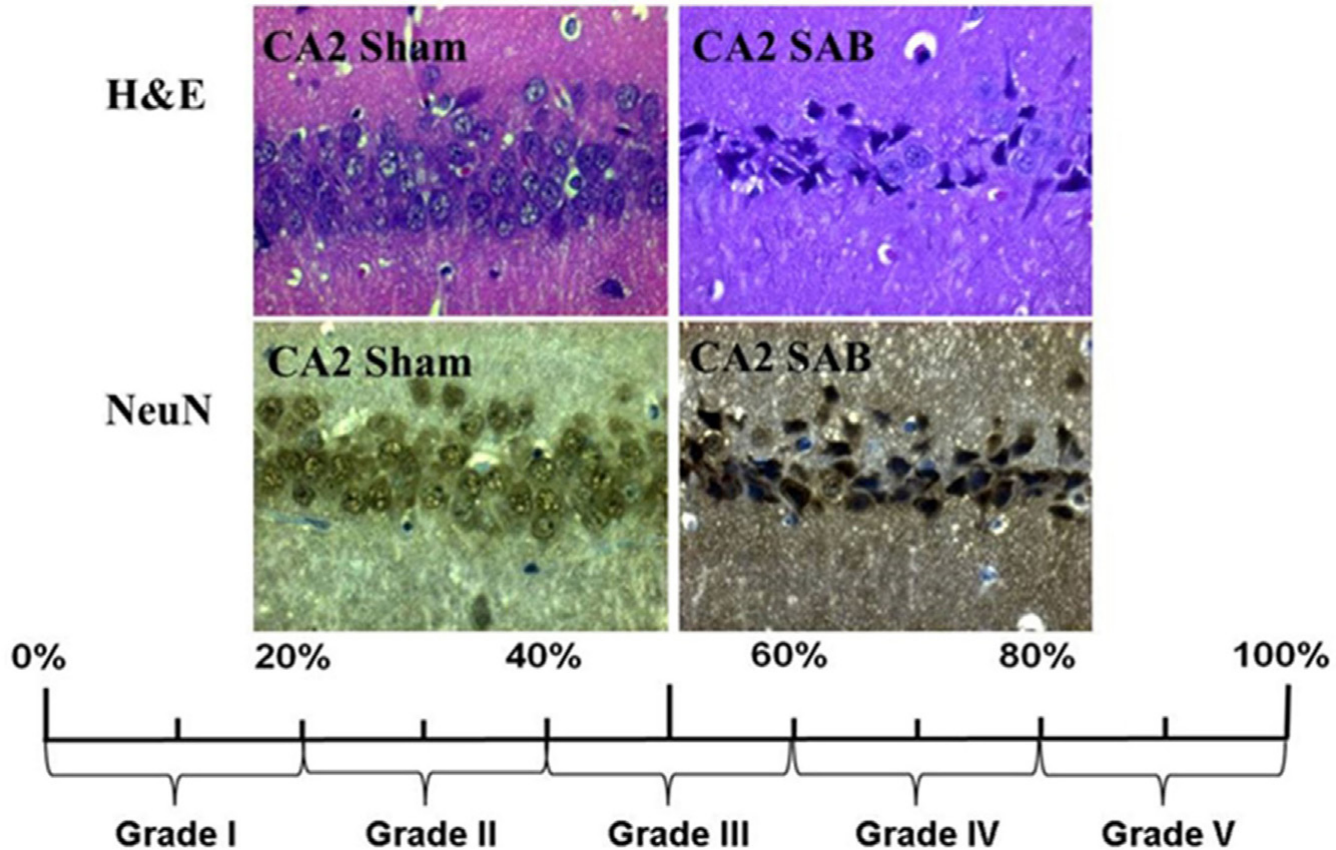
Histopathological severity score. Neuronal cell damage was evaluated in H&E and NeuN staining. Damaged neurons, characterized by hypereosinophilia, shrunken cytoplasm, and pyknotic nuclei, were software assisted counted and expressed as a ration to the total cell count per region. The resulting percentage was converted into grade 1 to grade 5.

Neuronal cell damage was measured and quantified in four regions of the left hippocampus, in H&E and NeuN-stained section: CA1, CA2, CA3, and dentate gyrus (DG). A single high power field (HPF) was focused on the center of each of these four region of interest (ROI) and the image was photographed with an Axioplan microscope (ZEISS, Germany). An absolute neuronal cell count as well as a cell count of all ischemic damaged neurons was done using ImageJ/Fiji v 1.50 (ImageJ Software downloaded at https://imagej.nih.gov/ij). See Figure 2. Neuronal cell damage was cytomorphologically defined as a combination of hypereosinophilia, shrunken cytoplasm, and pyknotic nuclei. This counting process was done twice by a single investigator blinded to treatment allocation on two different time-points and results were compared for incongruence. In case of incongruence or doubt, the consecutive NeuN stained slices was consulted and the process was repeated. The ratio of damaged neurons too the complete neuronal cell count was graded into five categories (1 = 0–20%, 2 = 20–40%, 3 = 40–60%, 4 = 60–80%, and 5 = 80–100%). See Figure 3. The results of this scale for each ROI in the left hemisphere were then summed to yield an overall neurohistopathological severity score per animal.

### Microglial Activation

An absolute microglial cell count was performed in a similar fashion in the Iba-1 (ionized calcium-binding adapter molecule 1) stained sections. Three cortical regions of interest per animal were photographed. The absolute number of activated Iba-1-positive cells was software-assisted counted out in the lateral primary somatosensory cortex (Pta), postero-lateral cortex (PLCo1), and the piriform cortex (Pir1) of both hemispheres. Figure 2 offers an overview of the selected regions of interest.

### Experimental Outcomes

The primary outcome was left side histopathological hippocampal damage as measured by our neurohistopathological severity score. The secondary outcome was microglial activation. In the initial trial design, a 24-h clinical evaluation using an 18- and 28-point scoring system was included. After the negative results in a similar study with argon, where we observed no difference in neurologic function in the acute phase (26), no short-term neurological evaluation was done in this trial.

### Statistical Methods

To estimate sample size data from previous experiments using argon as a neuroprotective agent were extrapolated (26). Using these data, an effect size of 0.62 was calculated (alpha: 0.1; beta: 0.8). As xenon is known to be more potent, we estimated an effect size of 0.7 (alpha: 0.1; beta: 0.8) resulting in a sample size of *n* = 6 (G*Power 3.9.1.2 downloaded at http://www.gpower.hhu.de/).

All statistical analyses were performed using SPSS v 23.0 (SPSS Inc., Chicago, IL, USA). All graphics were plotted using GraphPad Prism (GraphPad Software Inc., La Jolla, CA, USA). A *p*-value of <0.05 was considered statistically significant. After normality testing (Kolmogorov–Smirnov test), an unpaired *t*-test was used to analyze normally distributed numeric variables. Group comparisons were performed using one-way or two-way ANOVA testing followed by the appropriate *post hoc* test. All data are presented as means ± SD unless stated otherwise.

## Results

### Baseline Data

**Figure 4:**
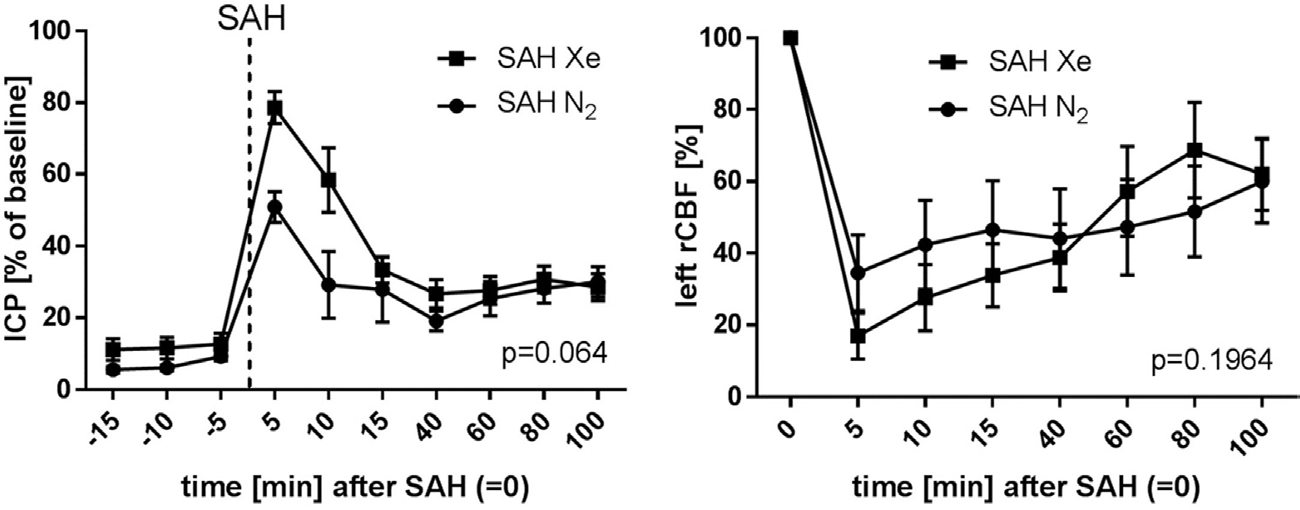
Course of ICP and rCBF. Graphs for intracranial pressure (ICP) and left regional cerebral blood flow (rCBF) are presented as mean ± SD of percentages of baseline values; time = 0 min is defined as subarachnoid hemorrhage (SAH). The courses did not differ between the xenon and control group (repeated measures analysis of variance).

The course of ICP and left side rCBF is illustrated in Figure 4. The SAH Xe group reached an overall higher peak in ICP increase after SAH induction compared to the control group. However, this difference was not statistically significant (*p* = 0.064). The initial decrease in rCBF after SAH induction did not differ significantly between the xenon and control group (*p* = 0.1964).

### Neuronal Cell Damage in H&E and NeuN-Stained Sections

**Figure 5:**
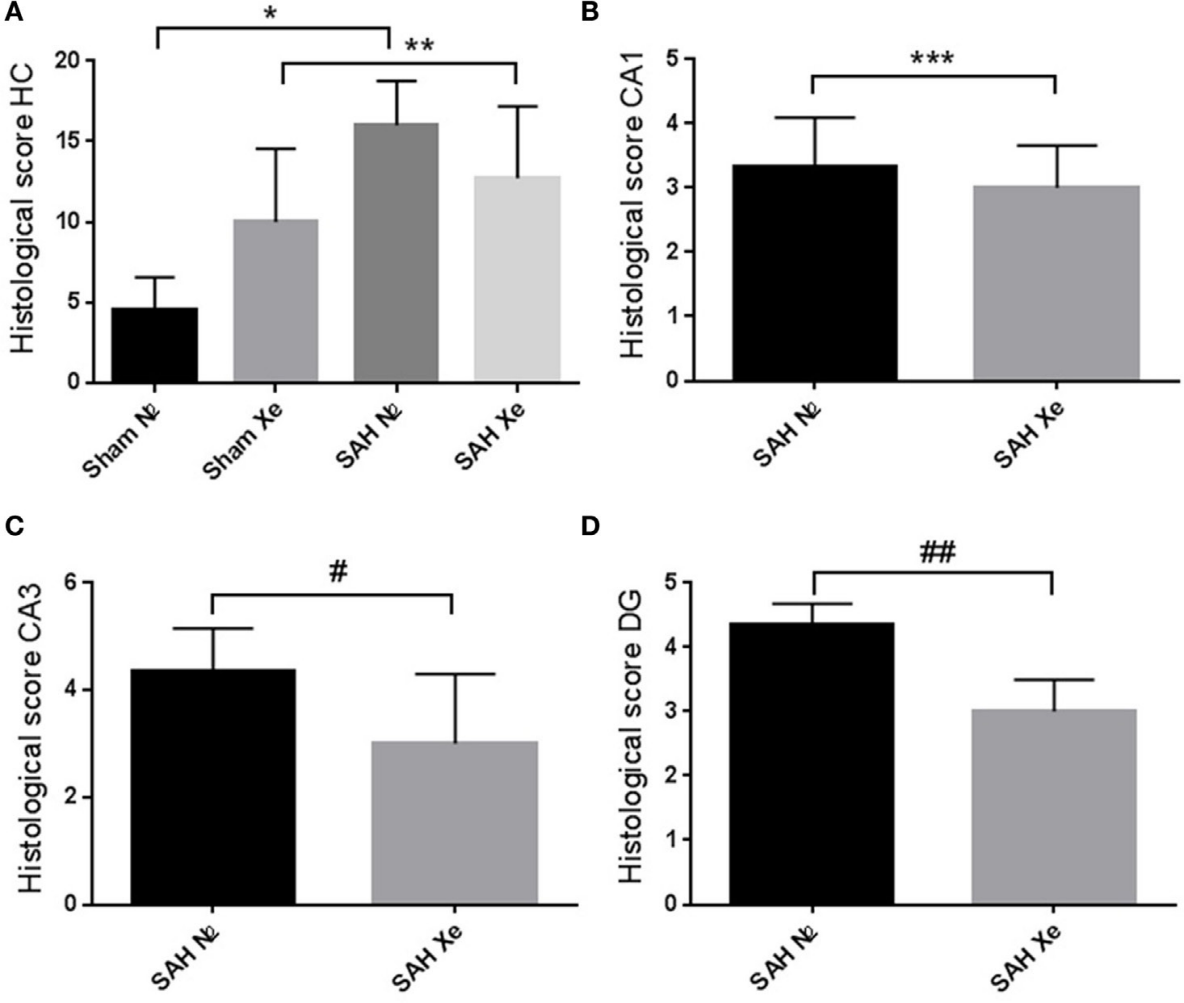
Hippocampal neuronal cell loss. Cytomorphological quantification of ischemic damage. (A) After summing up the four scores (0–5) per hippocampal region, a single score per animal between 0 and 20 was yielded. The summed-up scores were 4.5 ± 1.83 (Sham N_2_), 10 ± 4.05 (Sham Xe), 16 ± 2.52 (SAH N_2_), and 12.71 ± 4.13 (SAH Xe) group. There was no difference between the SAH N_2_ and SAH Xe groups. There was a significant difference between the Sham N_2_ and SAH N_2_ groups, *p = 0.0001. There was no significant difference between Sham Xe and SAH N_2_ groups, **p = 0.326. (B) The CA1 region of the SAH N_2_ group and the SAH Xe group had a mean histopathological severity score of 3.33 ± 1.70 vs. 3.0 ± 1.60, ***p = 0.4280. (C) In the CA3 regions, a significant difference group-score was seen, 4.5 ± 0.76 (SAH N_2_) vs. 2.71 ± 1.58 (SAH Xe) group, #p = 0.040. (D) The difference was also statistically significant in the DG regions 4.24 ± 1.38 vs. 3.09 ± 1.28, ##p = 0.040.

Routine hematoxylin and eosin staining was used to cytomorphologically quantify ischemic damage. Apoptotic neurons were clearly demarcated in the cell layers of all four assessed hippocampal regions. To compare treatment groups, the four scores (0–5) for each hippocampus were summed up to obtain a single score per animal between 0 and 20. Sham animals presented with mild baseline cell damage in all four hippocampal regions. The Sham N_2_ group had a summed-up score of 4.5 ± 1.83. The Sham Xe group presented with a summed-up score of 10 ± 4.05. The SAH N_2_ group had a summed-up score of 16 ± 2.52, and the SAH Xe group showed a summed-up score of 12.71 ± 4.13. These data are presented in Figure 5. The xenon-treated SAH group scored a lower summed-up score compared to the SAH N_2_ group, suggesting a protective effect of xenon after SAH. These differences proved, however, not to be statistically significant (*p* = 0.287). There was a significant difference between the Sham N_2_ and SAH N_2_ groups (*p* = 0.0001) illustrating the overall damage caused by the induced SAH. For the summed-up scoring, there was no significant difference between the Sham Xe and SAH Xe groups (*p* = 0.326). Once comparing individual hippocampal regions, it became apparent that the more medial located CA3 and DG regions were generally more intensely damaged compared to the more lateral located CA1 and CA2 regions. In those regions with more pronounced damaged the inter-group differences increased. Comparing the CA1 region of the SAH N_2_ group and the SAH Xe group, the mean histopathological severity score was 3.33 ± 1.70 vs. 3.0 ± 1.60 (*p* = 0.428). Whereas comparing the CA3 regions in the SAH N_2_ group, a mean score was yielded of 4.5 ± 0.76 vs. 2.71 ± 1.58 in the SAH Xe group (*p* = 0.040). The difference between the SAH N_2_ and SAH Xe group also became statistically significant in the DG regions 4.24 ± 1.38 vs. 3.09 ± 1.28 (*p* = 0.040).

### Microglial Activation in Iba-1 Stained Sections

**Figure 6:**
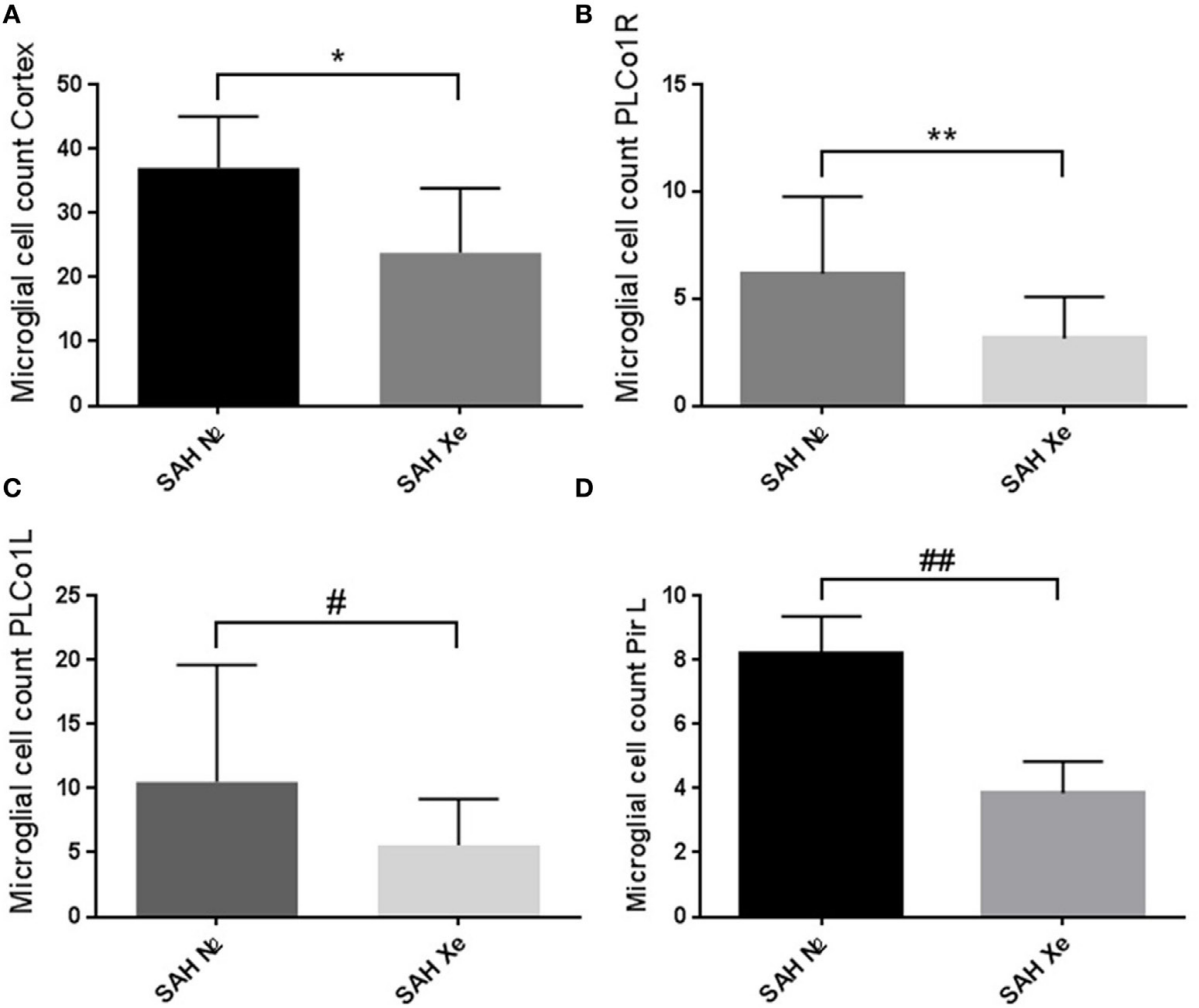
Cortical microglial cell count. Software assisted counting of Iba-1 positive cells in four cortical regions. **(A)** Comparing the summed up absolute cell count per animal, the xenon-treated animals showed a lower overall microglial cell count compared to the control groups, *p = 0.1206. **(B,C)** Regions closer to the bleeding source showed a higher number of activated microglia (highest damage in the PLCo1L region) with an increase in the difference between treatment groups without statistical significance, **p = 0.1680, #p = 0.1002. **(D)** The difference in cell count was statistically significant in the left side piriform cortex (pir1 L). 8 ±1.16 microglial cells were counted in the control group vs. 4 ± 1.07 in the xenon group, ##p = 0.017

In the brain, ionized calcium-binding adapter molecule 1 (Iba-1) is specifically expressed in microglia and, therefore, can be used as a robust biomarker for these cell types. Activated microglial cells are morphologically characterized by cellular branches and are easily identified using confocal microscopy. We determined the presence of activated microglia within three cortical regions for each hemisphere. Two regions are located at the base of the brain, close to the source of the induced hemorrhage (PLCo1 and Pir1). One region was chosen more distal to the bleeding source in the cranial parietal cortex (Pta). Iba-1-positive cells were software-assisted quantified in a HPF, focusing on the center of each ROI. In comparison to Sham-operated rats, SAH animals showed a clear increase in Iba-1-positive cells in all three cortical regions, reflecting the early inflammatory response after SAH. Once the absolute cell count of all three regions was summed up per animal, it became apparent that xenon-treated animals showed a lower overall microglial cell count compared to the control groups. See Figure 6. Also in all SAH animals, those regions closer to the initial bleeding source (left side Pir1 and PLCo1) showed a higher number of activated microglia reflecting the spatial distribution of severity of neuronal damage around the area of primary hit. This effect has been previously described in a rat SAH perforation model (27). In those regions of greater damage and microglial activation, the number of microglial cells was lower in the xenon-treated animals. This difference was statistically significant in the left side pir1 L where an average of 8 ± 1.16 microglial cells were counted per HPF in the control group vs. 4 ± 1.07 cells in the xenon group, *p* = 0.017.

## Discussion

We investigated the effects of xenon to attenuate EBI in a rat SAH model. Although a xenon-mediated protective effect was seen in all hippocampal regions, the protective effect of xenon was enhanced in the CA3 and DG regions. Second, we have shown a reduction in cortical microglial activity in xenon-treated animals. An intraparenchymal accumulation of microglia cells was more pronounced in regions closer to the site of vessel perforation. This effect has been previously demonstrated in animal as well as human tissue samples (28). In this trial by Schneider et al., a centrifugal spreading of microglia accumulation developed over time, from the base of the cortex of both hemispheres, resembling a wave of intracerebral immune cell activation. Similarly, we saw a gradual decrease of microglial activation in regions farther away from the site of primary hit. In the base of the left hemisphere, the regions with the highest accumulation of inflammatory cells, the immunomodulatory effect of xenon was the largest. Microglia plays a major role in the pro-inflammatory cytotoxic response and participates in the immunosuppressive processes contributing to further tissue damage (29). We postulate a possible immunomodulatory mechanism of xenon reducing microglial activation and contributing to a decrease in neuronal cell damage.

Although not significant (*p* = 0.168), it is unclear why the Sham Xe group presented a higher summed-up score and thus more hippocampal damage, compared to the Sham N_2_ group. It could be that Xenon has no effect on the background damage occurring in our Sham animals indicating that the neuroprotective effect of Xenon cannot be generalized.

We have previously demonstrated a reduction in mortality after argon post-conditioning in a SAH animal model (26). Here, we present for the first time the beneficial effect of xenon treatment after experimental SAH with a reduction of hippocampal neuronal cell loss and a decrease in cortical microglial cell activity. Until now, the neuroprotective effects of xenon have been accredited to the inhibition of the NMDA receptor (30–32). *In vitro* research has shown that NMDA receptor stimulation triggers microglia activation and the secretion of neurotoxic factors (33). This could very well explain the immunomodulatory mechanism observed in our xenon-treated animals. Xenon may be a potential clinical treatment for EBI under carefully defined conditions. By attenuation of the complex inflammatory mechanisms, some of the devastating secondary injuries may be prevented and outcome in SAH patients could be improved.

### Limitations

The primary weakness of our trial was the limited number of included animals. We estimated that a higher number of included animals could demonstrate a more pronounced therapeutic effect. Second, per ROI, only a single HPF was evaluated for calculation of neuronal cell loss (in H&E and NeuN Staining) and for the evaluation of microglial activation in Iba-1 staining. In the initial trial design, neuronal cell loss was manually counted in the entire hippocampus. As the trial continued, we observed a good congruence between these results and the estimated damage using only a single HPF. Additionally, to estimate neuronal cell loss, we only looked at the right hippocampus. This was done because the insertion of the ICP probe on the left side caused additional damage to the left hemisphere introducing potential bias. ROIs in the left hemisphere were included for the assessment of microglial activation since they were located at the base of the brain, farther away from the regions of iatrogenic damage.

Until now, we did not examine whether the xenon-mediated reduction in Iba-1-positive cells coincided with a reduction in cortical damage. Because of its higher cell count, estimating cortical damage is more time consuming but could be the focus of future research projects. Additionally, it is worth mentioning that Iba-1 is not a specific marker for microglia and unspecific binding of the antibody could have compromised our results.

## Conclusion

This is the first time that a neuroprotective effect of xenon has been shown. The effect is potentially mediated by an inhibitory effect on microglial activation. Further animal research should focus on long-term clinical outcome post Xenon ventilation in a SAH model.

## Ethics Statement

The study protocol was approved by the government agency for animal use and protection (Protocol number: TVA 10416G1 approved by “Landesamt für Natur, Umwelt und Verbraucherschutz NRW,” Recklinghausen, Germany), all experiments were conducted in accordance with the Guide for Care and Use of Laboratory Animals (National Research Council, and the Committee for the Update of the Guide for the Care and Use of laboratory Animals; 8th edition 2011).

## Author Contributions

Animal experiments: AH. Data analysis: MV and AH. Manuscript drafting: MV. Manuscripts revision and editing: MV, MC, RR, HC, BK, and AH. Final approval of the manuscript: MV, MC, RR, HC, BK, and AH.

## Conflict of Interest Statement

AH lectured for Air Liquide. RR consulted and lectured for Baxter Healthcare and Air Liquide. His institution received grant Support from the Deutsche Forschungsgemeinschaft, Baxter Healthcare, and Air Liquide. All remaining authors have no potential conflict of interest to declare.

## Abbreviations

SAH, subarachnoid hemorrhage; ICP, intracranial pressure; rCBF, regional cerebral blood flow; ROI, region of interest; ICA, internal carotid artery; HPF, high power field.
